# Influence of pharmacogenomic polymorphisms on allopurinol-induced cutaneous adverse drug reactions in Thai patients

**DOI:** 10.1186/s12920-024-01874-y

**Published:** 2024-04-23

**Authors:** Gaidganok Sornsamdang, Patompong Satapornpong, Pimonpan Jinda, Thawinee Jantararoungtong, Napatrupron Koomdee, Therdpong Tempark, Jettanong Klaewsongkram, Ticha Rerkpattanapipat, Pawinee Rerknimitr, Papapit Tuchinda, Leena Chularojanamontri, Napatra Tovanabutra, Kumutnart Chanprapaph, Wareeporn Disphanurat, Panlop Chakkavittumrong, Chutika Srisuttiyakorn, Yuttana Srinoulprasert, Shobana John, Mohitosh Biswas, Chonlaphat Sukasem

**Affiliations:** 1 Division of Pharmacogenomics and Personalized Medicine, Department of Pathology, Faculty of Medicine Ramathibodi Hospital, Mahidol University, Bangkok, Thailand; 2 Laboratory for Pharmacogenomics, Somdech Phra Debaratana Medical Center (SDMC), Ramathibodi Hospital, Bangkok, Thailand; 3 Division of General Pharmacy Practice, Department of Pharmaceutical Care, College of Pharmacy, Rangsit University, Pathum Thani, Thailand; 4 Excellence Pharmacogenomics and Precision Medicine Centre, College of Pharmacy, Rangsit University, Pathum Thani, Thailand; 5 Division of Pediatric Dermatology, Department of Pediatrics, Faculty of Medicine, Chulalongkorn University, Bangkok, Thailand; 6 Division of Allergy and Clinical Immunology, The Skin and Allergy Research Unit, Department of Medicine, Faculty of Medicine, Chulalongkorn University, Bangkok, Thailand; 7 Division of Allergy Immunology and Rheumatology, Department of Medicine, Faculty of Medicine, Ramathibodi Hospital, Mahidol University, Bangkok, Thailand; 8 Division of Dermatology, The Skin and Allergy Research Unit, Department of Medicine, Faculty of Medicine, Chulalongkorn University, Bangkok, Thailand; 9 Department of Dermatology, Faculty of Medicine Siriraj Hospital, Mahidol University, Bangkok, Thailand; 10 Dermatological Division, Department of Internal Medicine, Chiang Mai University, Chiang Mai, Thailand; 11 Division of Dermatology, Department of Medicine, Faculty of Medicine, Ramathibodi Hospital, Mahidol University, Bangkok, Thailand; 12 Division of Dermatology, Department of Medicine, Faculty of Medicine, Thammasat University, Pathumthani, Thailand; 13 Division of Dermatology, Department of Medicine, Phramongkutklao Hospital, Phramongkutklao College of Medicine, Bangkok, Thailand; 14 Department of Immunology, Faculty of Medicine Siriraj Hospital, Mahidol University, Bangkok, Thailand; 15 Department of Pharmacy, University of Rajshahi, 6205 Rajshahi, Bangladesh; 16 The Thai Severe Cutaneous Adverse Drug Reaction (THAI-SCAR) Research Group, Bangkok, Thailand; 17 Pharmacogenomics Clinic, Bumrungrad Genomic Medicine Institute, Bumrungrad International Hospital, 10110 Bangkok, Thailand; 18 Research and Development Laboratory, Bumrungrad International Hospital, Bangkok, Thailand; 19 Faculty of Pharmaceutical Sciences, Burapha University, 20131 Saensuk, Mueang, Chonburi Thailand; 20 Department of Pharmacology and Therapeutics, MRC Centre for Drug Safety Science, Institute of Systems, Molecular, and Integrative Biology, University of Liverpool, Liverpool, UK

**Keywords:** Allopurinol, Pharmacogenomics, Single-nucleotide polymorphisms, Human leucocyte antigen, Cutaneous adverse drug reactions

## Abstract

**Background:**

Allopurinol has been causing substantial morbidity and mortality particularly in Asian population by producing cutaneous adverse drug reactions (cADRs). Nonetheless, there are no data describing whether other genetics are a valid marker for prediction of allopurinol-induced cADRs patients in addition to HLA-B*58:01 allele. The goal of this study was to identify suitable single nucleotide polymorphisms (SNPs) for allopurinol induced cADRs among Thai patients. Methods: We conducted a case-control association study after enrolling 57 Thai patients with allopurinol induced cADRs and 101 allopurinol-tolerant controls. The genetic biomarkers and associated SNPs located on chromosome 6p21 were examined by TaqMan® SNP genotyping assays in both the cases and the controls.

**Results:**

Out of fifteen SNPs in nine genes, we found four combined SNPs (*rs3099844* of *HCP5*, *rs9263726* of *PSORS1C1*, *rs9263733* of *POLR2LP*, and *rs9263745* of *CCHCR1*) were significantly associated with allopurinol-induced cADRs compared to the tolerant controls (OR 73.2; 95% CI 24.2–266.8; *P* = 1.9 × 10^− 24^). The overall sensitivity, specificity, positive predictive value and negative predictive value of these combinations were 84%, 94%, 9%, and 100%, respectively. However, the variant alleles of these SNP combinations were detected in 89.5% (51/57) of the cases. Moreover, the HLA-B*58:01 allele was observed in 86.0% of patients with allopurinol-induced cADRs, but only in 4.0% of tolerant controls (OR: 137.2; 95% CI: 38.3–670.5 and *p*-value = 1.7 × 10^− 27^).

**Conclusions:**

Thus, this research confirms the association between the specific HLA-B*58:01 allele and all phenotypes of allopurinol-induced cADRs in Thais. Furthermore, there was found the combined four *SNPs* (*rs3099844, rs9263726, rs9263733*, and *rs9263745*) could be used as alternative novel biomarkers for predicting cADRs in patients taking allopurinol.

**Supplementary Information:**

The online version contains supplementary material available at 10.1186/s12920-024-01874-y.

## Introduction

Allopurinol, a xanthine oxidase inhibitor involved in purine catabolism, is commonly prescribed in patients who have hyperuricemia with gouty arthritis, and it can be used prophylactically to prevent chemotherapy induced hyperuricemia [1, 2]. Cutaneous adverse drug reactions (cADRs) associated with allopurinol can cause substantial morbidity and mortality in these patients, particularly those of Asian ethnicities [3]. The cADRs, which involve delayed immune-mediated mechanisms, present with different clinical patterns that have been very well characterized phenotypically and they include urticarial rash, Stevens - Johnson syndrome (SJS), toxic epidermal necrolysis (TEN), drug reactions with eosinophilia and systemic symptoms (DRESS) and maculopapular exanthema (MPE) [4]. In Thai population, HLA-B*58:01 has been significantly associated with allopurinol-induced severe cutaneous adverse drug reactions (SCARs), i.e., SJS/TEN (odds ratio (OR) of 348.30; 95% confidence interval (CI) 19–6336; *P* = 1.6 × 10^− 13^) [2] and it has also been significantly associated with cADRs (OR 696.00; 95% CI 74–6475; *P* < 0.01) [5]. Numerous studies have reported strong associations between the HLA-B*58:01 allele and allopurinol-induced SJS, TEN, DRESS and MPE in other ethnic groups. For example, past studies have achieved ORs of 580.30 (*P* = 4.7 × 10^− 24^; 95% CI 34–9780) for Han-Chinese patients [1], 61.00 (*P* = 1.0 × 10^− 8^; 95% CI 32–118) for European patients [6], 65.60 (*P* = 9.73 × 10^− 4^; 95% CI 2.9–1497.0) for Japanese patients [7], 97.80 (*P* = 2.4 × 10^− 11^; 95% CI 18.3–521.5) for Korean patients [8], 13.60 (*P* = 0.003; 95% CI 2.77–69.45) for Caucasian patients [9], and 39.11 (*P* < 0.01; 95% CI 4–340) for Portuguese patients [10]. These results suggest that HLA-B*58:01 could be considered a genetic biomarker for allopurinol-induced cADRs or SCARs, without having to specify ethnic differences.

However, the results from the study by Saksit N. et al. [11] mentioned that “three single nucleotide polymorphisms (SNPs) including *rs9263726*, *rs2734583*, and *rs3099844* were significantly associated with allopurinol-induced SCARs but with a lower degree of association when compared with the HLA-B*58:01 allele.” The sensitivity, specificity, PPV, and NPV of these SNPs were comparable to those of the HLA-B*58:01 allele. Although detection of the SNP is simpler and less expensive compared with detecting the HLA-B*58:01 allele, these SNPs were not perfectly linked with the HLA-B*58:01 allele. Moreover, some of the Thai patients experienced allopurinol-induced cADRs even though they did not harbor the HLA-B*58:01 allele [5].

A genome-wide association study (GWAS) investigating the genetic associations of SJS/TEN in a large sample of European patients found significant associations for six SNPs located in the *HLA* region (OR:1.53–1.74), including the *HCP5 (rs9469003), PSORS1C1 (rs3815087)*, and *POU5F1 (rs3130931, rs3130501*, and *rs3094188)* genes. The haplotype formed by their risk allele was strongly associated with allopurinol-induced SJS/TEN more than any of the other single SNPs (OR:7.77; 95% CI 4.66–12.98; *P* = 6.56 × 10^− 7^). Although the associated haplotype was in linkage disequilibrium with the HLA-B*58:01 allele, the findings of this GWAS provided insightful information regarding the potential association of allopurinol-induced SJS/TEN with other possible genes e.g. *HCP5*, *PSORS1C1* and *POU5F1*, in addition to *HLA-B* [12].

Another GWAS conducted on Japanese patients showed that a total of 21 SNPs located on 6p21 were significantly associated with allopurinol-induced SJS/TEN after analyzing a total of 890,321 SNPs, and the strongest associations were found for the *BAT1* (*rs2734583*) and *HCP5* (*rs3094011*) genes (OR 66.8; 95% CI 19.8–225.0; *P* = 2.44 × 10^− 8^). The study also established a significant association between *PSORS1C1* (*rs9263726*) and allopurinol-induced SJS/TEN, although *PSORS1C1 (rs9263726*) was in absolute linkage disequilibrium with HLA-B*58:01, but this still suggested that *PSORS1C1* could be an alternative biomarker for predicting allopurinol-induced SJS/TEN in Japanese patients because this SNP is easy to genotype [13]. A recent study conducted in Australia found no linkage disequilibrium for *PSORS1C1* (*rs9263726*) and the HLA-B* 58:01 allele, and it indicated that *PSORS1C1* (*rs9263726*) could not be used as a surrogate biomarker to identify carriers for the HLA-B*58:01 allele [14]. Furthermore, a very recent study reported a significant association between two SNPs of *TCF19* (*rs9263794* and *rs1044870*) and one SNP of *POU5F1* (*rs9263796*) and allopurinol-induced cADRs in Thai patients (OR:57.20, *P* < 0.001 for *rs9263794*; OR:77.31, *P* < 0.001 for *rs1044870*; OR:84.14, *P* < 0.001 for *rs9263796*), but it did not specify whether these SNPs were in linkage disequilibrium with the HLA-B*58:01 allele or if they could be used as alternative biomarkers for predicting allopurinol-associated cADRs [15]. Consequentially, new research was warranted to assess whether the genes of interest may have novel SNPs that could be considered as potential alternative biomarkers for predicting allopurinol-induced cADRs.

Therefore, this study aimed to investigate the associations of the SNPs in the genes of interest i.e. *BAT1*, *BAT3*, *HCP5*, *PSORS1C1*, *POLR2LP*, *CCHCR1*, *TCF19*, *POU5F1*, *HLA-C* and *MSH5* located in 6p21 with allopurinol-induced cADRs in Thai patients, in addition to the formal typing of HLA-B*58:01 allele.

## Materials and methods

### Recruitment of study subjects

We carried out a case-control study to investigate the association of a number of genes of interest with allopurinol-induced cADRs where the patients were recruited retrospectively and prospectively. Fifty-seven patients with allopurinol-induced cADRs from the Thai Severe Cutaneous Adverse Drug Reaction (THAI-SCAR) project and patients admitted to the Allergy Clinic of the Faculty of Medicine, Ramathibodi Hospital, Mahidol University were enrolled. Consequently, 57 subjects were categorized into SJS/TEN (25 cases), DRESS (24 cases), and MPE (8 cases). Patients who had been taking allopurinol for more than 6 months with no evidence of cADRs were recruited as the allopurinol-tolerant controls (*n* = 101). This study was performed with approval from the Ramathibodi Hospital’s ethical review board, and informed consent was obtained from all of the participants (MURA2015/300).

All patients with cADRs were assessed by a dermatologist and an allergist independently. The phenotypes were classified using the RegiSCAR criteria [16]. Patients were diagnosed with DRESS if they had fevers, skin rashes, hematologic abnormalities (e.g., eosinophilia or atypical lymphocytes), enlarged lymph nodes and internal organ involvement (e.g., liver, kidney, lung, or heart muscle). Patients were diagnosed with SJS and TEN if they had skin rashes and mucosal detachment where SJS was defined as having a body surface area (BSA) skin detachment of less than 10%, TEN was defined as having more than a 30% BSA skin detachment, and an SJS/TEN overlap was defined as having a 10–29% BSA skin detachment. MPE was characterized by the presence of danger signs in drug-induced exanthema with or without associated systematic symptoms but not reaching the criteria for DRESS [17]. The culprit drugs causing the cADRs were determined using the Naranjo algorithm [18].

### Genomic DNA extraction

Blood samples of 3–6 ml were collected from the patients into EDTA-treated tubes. DNA extraction was performed using MagNA Pure Compact (Roche Applied Science, Pennsburg, Germany). The quality of the genomic DNA was assessed using a Nano Drop, ND-1000. Genomic DNA was stored at 2–8 °C for up to one week or frozen at -20 °C for up to one month with no freeze-thaw cycles before analysis.

### SNP genotyping assay

Fifteen SNPs, *rs2734583* (A > G; *BAT1*), *rs3099844* (C > A; *HCP5*), *rs9263726* (G > A; *PSORS1C1*), *rs2233945* (C > A; *PSORS1C1*), *rs9263733* (C > T; *POLR2LP*), *rs9263745* (G > A; *CCHCR1*), *rs130077* (G > A; *CCHCR1*), *rs9263785* (T > G; (G > A; *CCHCR1*), *rs9263794* (A > G; *TCF19*), *rs1044870* (C > T; *TCF19*), *rs9263796* (C > T; *POU5F1*), *rs4084090* (A > G; *HLA-C*), *rs3131643* (G > A; *HCP5*), *rs3117583* (A > G; *BAT3*), and *rs1150793* (A > G; *MSH5*) in nine genes were assayed in a ninety-six-well plate using a TaqMan real-time PCR Viia7 (ABI, Foster City CA, USA) following the manufacturer’s instructions.

### Genotyping of the HLA-B*58:01 allele

The genotyping method for *HLA-B* alleles was reported and described elaborately in our previous investigation [5]. In short, *HLA-B* typing was undertaken following the principle of the PCR-sequence specific oligonucleotide probe (PCR-SSOP) with a commercially available kit. Polymerase chain reaction (PCR) was used to amplify the diluted DNA samples using a GeneAmp®PCR System9700 (Applied Biosystems, Waltham, USA). Genotyping of the specific HLA-B*58:01 alleles was carried out using Luminex™ multiplex technology (Luminex® IS 100, USA) by hybridizing the PCR product against a panel of oligonucleotide probes having specific sequence targeting the HLA-B*58:01 allele and finally the detection was confirmed by visualization following standard protocols [5]. MATCH IT DNA software version 3.2.1 was a powerful tool for analysis of the HLA alleles (One Lambda, Canoga Park, CA, USA).

### Statistical analysis

All variant candidate SNPs were tested for association using a chi-square test or Fisher’s exact test (OR; 95% CI) using R version 3.1.1. The ORs were calculated using the MEDCALC® program. *P*-values were corrected for multiple testing according to Bonferroni’s correction. *P*-values less than 3.3 × 10^− 3^ were considered statistically significant. The sensitivity, specificity, negative predictive value (NPV) positive predictive value (PPV) were calculated using the percentages from the variant alleles in each of the SNPs.

## Results

### Characteristics of the study subjects

Of the 57 patients with allopurinol-induced cADRs, 29 (51%) were female, and 28 (49%) were male, and the mean age was 66 years. The mean duration of allopurinol use was 21 days. Of the 57 patients with allopurinol-induced cADRs, 49 patients carried the HLA-B*58:01 allele (85.9%), and 8 patients that did not carry the HLA-B*58:01 allele were categorized as follows: 1 SJS/TEN case (1.75%), 5 DRESS cases (8.77%) and 2 MPE cases (3.51%) (Table 1). The allopurinol exposure period was 21 days. The average allopurinol dose was 159 ± 109 mg/day. The underlying diseases identified in the greatest numbers of patients were hypertension (27; 47.4%) followed by chronic kidney disease (18;31.6%), diabetes (13;22.8%), and dyslipidemia (4;7.0%). Out of the 57 allopurinol-induced cADR patients, 20 (35.1%) were using colchicine and prednisolone as co-medications.

**Table 1:** Demographic data of the allopurinol-induced cutaneous adverse drug reactions (cADRs)

Demographic Data	Case (*n* = 57)	Control (*n* = 101)	P-value
Sex, n (%)			
Female	29 (51)	78 (77)	< 0.001
Male	28 (49)	23 (23)	
Age (years)			
Mean	66	62	0.087
Median	69	64	
Phenotype, n (%)			
SJS-TEN	25 (43.9)	-	
DRESS	24 (42.1)	-	
MPE	8 (14.0)	-	
Allopurinol exposure			
Duration used (days)	21	776	< 0.001
Dose/day (mg)			
Mean (average ± SD)	159 ± 109	158 ± 105	0.992
Median (range)	100	100	
Laboratory result (mean ± SD)			
BUN, mg/dl	48 ± 32	22 ± 8	< 0.001
Creatinine, mg/dl	1.96 ± 1.43	1.72 ± 1.22	0.174
AST, U/L	99 ± 129	30 ± 20	< 0.001
ALT, U/L	123 ± 152	37 ± 25	< 0.001
Underlying disease, n (%)			
Hypertension	27 (47.4)	60 (59.4)	0.878
Chronic kidney disease	18 (31.6)	33 (32.7)	0.361
Diabetes	13 (22.8)	13 (12.9)	0.017
Dyslipidemia	4 (7.0)	16 (15.8)	0.017
Cancer	0	6 (5.9)	-
Co-medication, n (%)			
Colchicine	20 (35.1)	52 (51.5)	0.469
Prednisolone	2 (3.5)	9 (8.9)	0.354
Simvastatin	5 (8.8)	16 (15.8)	0.659
Sodium bicarbonate	3 (5.3)	16 (15.8)	0.134

*Abbreviations* ALT, alanine Aminotransferase; AST, aspartate amino transferase; BUN, blood urea nitrogen; Stevens-Johnson syndrome, SJS; toxic epidermal necrolysis, TEN; drug reaction with eosinophilia and systemic symptoms, DRESS and maculopapular exanthema, MPE

### Association between HLA-B*58:01 allele and SNPs with allopurinol-induced cADRs

The phenotypic variances among the allopurinol-induced cADRs with the SNP distributions are described in Table 2. As shown in Table 3, *rs9263733* of *POLR2LP* was the most significant SNP associated with allopurinol-induced cADRs, with an OR of 96.1 (95%CI 29.4-390.8, *P*-value 1.4 × 10^− 25^). This variant was detected in 84.2% (48/57) of the cases and 4.9% (5/101) of the tolerant controls. The percentages for sensitivity, specificity, PPV, and NPV are shown in Supplement 1. The variant *rs9263745* contained in the *CCHCR1* gene was significantly associated with allopurinol-induced cADRs, with an OR of 77.7 (95% CI 25.5–276.41; *P*-value = 1.1 × 10^− 24^). It was detected in 85.9% (49/57) of the cases and 6.9% (7/101) of the tolerant controls. The next highest significant associations for allopurinol-induced cADRs were observed in four SNPs (*rs9263785*, *rs130077*, *rs9263726*, and *rs9263796*), each with an OR of 67.8 (95% CI 22.9–234.9; *P*-value = 1.1 × 10^− 23^). They were detected in 84.2% (48/57) of the cases and 6.9% (7/101) of the tolerant controls. As described in Table 3, *rs9263733* (*POLR2LP*) was the most significantly associated SNP with allopurinol-induced SJS/TEN, with an OR of 395.2 (95% CI 49.0–16306.3; *P*-value = 7.1 × 10^− 20^). The next highest associations were found for *rs9263745 (CCHCR1)*, *rs9263785 (CCHCR1)*, *rs9263796 (POU5F1)*, *rs9263726 (PSORS1C)* and *rs130077 (CCHCR1)*, each with an OR of 287.6 (95% CI 37.6–12034.6; *P*-value = 1.5 × 10^− 8^). The SNP *rs9263733* (*POLR2LP*) was also associated with allopurinol-induced DRESS with an OR of 66.9 (95% CI 16.6–345.1; *P*-value = 1.1 × 10^− 13^). The SNP *rs9263745* (*CCHCR1*) was associated with allopurinol-induced MPE, with an OR of 37.2 (95% CI 5.4–436.9, *P*-value = 2.1 × 10^− 5^).

**Table 2:** Distribution of the genetic variants among the cases and controls

HLA/SNPs allele	Tolerant control (*n* = 101) n (%)	cADRs (*n* = 57) n (%)	SJS/TEN (*n* = 25) n (%)	DRESS (*n* = 24) n (%)	MPE (*n* = 8) n (%)
HLA-B*5801	4 (3.9)	49 (85.9)	24 (96.0)	19 (79.2)	6 (75.0)
*BAT1* rs2734583, A > G	7 (6.9)	44 (77.2)	22 (88.0)	19 (79.2)	3 (37.5)
*BAT3* rs3117583, A > G	16 (15.8)	38 (66.7)	20 (80.0)	14 (58.3)	4 (50.0)
*CCHCR1* *rs130077, G > A*	7 (6.9)	48 (84.2)	24 (96.0)	19 (79.1)	5 (62.5)
*CCHCR1* *rs9263745, G > A*	7 (6.9)	49 (85.9)	24 (96.0)	19 (79.1)	6 (75.0)
*CCHCR1* *rs9263785, T > G*	7 (6.9)	48 (84.2)	24 (96.0)	19 (79.1)	5 (62.5)
*HCP5* *rs3099844, C > A*	8 (7.9)	45 (78.9)	22 (88.0)	20 (83.3)	4 (50.0)
*HCP5* *rs3131643, G > A*	15 (14.9)	46 (80.7)	22 (88.0)	20 (83.3)	3 (37.5)
*HLAC* *rs4084090, A > G*	11 (10.9)	49 (85.9)	24 (96.0)	20 (83.3)	5 (62.5)
*MSH5* rs1150793, A > G	18 (17.8)	38 (66.7)	19 (76.0)	14 (58.3)	4 (50.0)
*POLR2LP* rs9263733, C > T	5 (4.9)	48 (84.2)	24 (96.0)	19 (79.2)	5 (62.5)
*POU5F1* rs9263796, C > T	7 (6.9)	48 (84.2)	24 (96.0)	19 (79.1)	5 (62.5)
*PSORS1C1* *rs2233945, C > A*	7 (6.9)	47 (82.5)	24 (96.0)	18 (75.0)	5 (62.5)
*PSORS1C1* *rs9263726, G > A*	7 (6.9)	48 (84.2)	24 (96.0)	19 (79.2)	5 (62.5)
*TCF19* rs1044870, C > T	0 (0)	14 (24.6)	2 (8.0)	8 (33.3)	4 (50.0)
*TCF19* rs9263794, A > G	12 (11.9)	49 (85.9)	24 (96.0)	20 (83.3)	5 (62.5)

*Abbreviations* cutaneous adverse drug reactions, cADRs; Stevens-Johnson syndrome, SJS; toxic epidermal necrolysis, TEN; drug reaction with eosinophilia and systemic symptoms, DRESS; maculopapular exanthema, MPE and single nucleotide polymorphisms, SNPs

We compared the genotype frequency of HLA-B*58:01 alleles were 49 of 57 (85.9%) in allopurinol-induced cADRs, 4 of 101 (3.96%) in tolerant control as shown in Table 2. Interestingly, HLA-B*58:01 showed a strongly associated with allopurinol-induced cADRs in Thai patients (OR = 137.2; 95%CI = 38.3–670.5 and pc-value = 1.7 × 10^− 27^). Moreover, we found that the 96.0%, 79.2% and 75% of allopurinol-induced SJS/TEN, DRESS and MPE cases carried HLA-B*58:01 allele, respectively. This study showed that the HLA-B*58:01 associated with allopurinol-induced SJS/TEN (odds ratio = 582.0; 95% CI: 62.2–5447.3; pc-value = 3.7 × 10^− 23^), DRESS (odds ratio = 92.2; 95% CI: 22.6–375.1; pc-value = 2.3 × 10^− 14^) and MPE (odds ratio = 63.7; 95% CI: 8.4–829.2; pc-value 2.7 × 10^− 6^) (Table 3). Particularly, sensitivity, specificity, PPV and NPV of HLA–B*58:01 allele for prediction of allopurinol-induced cADRs were 86%, 96%, 92% and 92%, respectively (Supplement 3). Although, in this study was found the eight allopurinol-induced cADRs patients without HLA-B*58:01 allele. However, only one of the eight cases was positive for the combined 4 SNPs (Supplement 2).

**Table 3:** The Association between HLA-B*58:01 allele and SNPs with allopurinol-induced cADRs

HLA/SNPs allele	cADRs vs. tolerant control		SJS/TEN vs. tolerant control		DRESS vs. tolerant control		MPE vs. tolerant control	
	Odds ratio (95% CI)	P-value	Odds ratio (95% CI)	P-value	Odds ratio (95% CI)	P-value	Odds ratio (95% CI)	P-value
HLA-B*58:01	137.2 (38.3–670.5)	1.7 × 10^− 27^	582.0 (62.2–5447.3)	3.7 × 10^− 23^	92.2 (22.6–375.1)	2.3 × 10^− 14^	63.7 (8.4–829.2)	2.7 × 10^− 6^
*BAT1* rs2734583, A > G	43.5 (15.6–140.6)	4.4 × 10^− 20^	90.1 (20.7–580.1)	1.4 × 10^− 15^	47.6 (12.8–219.8)	1.5 × 10^− 12^	7.8 (1.0–51.0)	2.5 × 10^− 2^
*BAT3* rs3117583, A > G	10.4 (4.6–24.7)	1.8 × 10^− 10^	20.5 (6.3–80.3)	2.07 × 10^− 9^	7.3 (2.5–22.0)	5.2 × 10^− 5^	5.2 (0.9–31.1)	3.6 × 10^− 2^
*CCHCR1* *rs130077, G > A*	67.8 (22.9–234.9)	1.1 × 10^− 23^	287.6 (37.6–12034.6)	1.5 × 10^− 18^	47.6 (12.8–219.8)	1.5 × 10^− 12^	20.9 ( 3.3–164.6)	3.2 × 10^− 4^
*CCHCR1* *rs9263745, G > A*	77.7 (25.5–276.4)	1.1 × 10^− 24^	287.6 (37.6–12034.6)	1.5 × 10^− 18^	47.6 (12.8–219.8)	1.5 × 10^− 12^	37.2 (5.4–436.9)	2.1 × 10^− 5^
*CCHCR1* *rs9263785, T > G*	67.8 (22.9–234.9)	1.1 × 10^− 23^	287.6 (37.6–12034.6)	1.5 × 10^− 18^	47.6 (12.8–219.8)	1.5 × 10^− 12^	20.9 ( 3.3–164.6)	3.2 × 10^− 4^
*HCP5* *rs3099844, C > A*	41.8 (15.3–129.7)	3.7 × 10^− 20^	40.1 (10.3–234.6)	6.7 × 10^− 12^	27.5 (7.8–126.1)	2.7 × 10^− 10^	5.6 (0.9–33.6)	2.9 × 10^− 2^
*HCP5* *rs3131643, G > A*	23.3 (9.5–62.3)	2.1 × 10^− 16^	78.7 (18.5–495.9)	5.2 × 10^− 15^	54.2 (14.0–275.4)	3.4 × 10^− 13^	6.8 (0.9–43.1)	3.2 × 10^− 2^
*HLA-C* *rs4084090, A > G*	47.9 (17.3–150.3)	1.1 × 10^− 21^	181.9 (25.3–7691.2)	2.4 × 10^− 16^	38.7 (10.6–185.4)	8.2 × 10^− 12^	13.0 (2.2–95.7)	1.6 × 10^− 3^
*MSH5* *rs1150793, A > G*	9.1 (4.1–20.9)	1.2 × 10^− 9^	16.7 (5.3–64.5)	1.7 × 10^− 8^	5.9 (2.1–17.5)	2.2 × 10^− 4^	4.2 (0.7–24.9)	5.9 × 10^− 2^
*POLR2LP* *rs9263733, C > T*	96.1 (29.4–390.8)	1.4 × 10^− 25^	395.2 (49.0–16306.3)	7.1 × 10^− 20^	66.9 (16.6–345.1)	1.1 × 10^− 13^	29.4 (4.3–247.9)	1.06 × 10^− 4^
*POU5F1* *rs9263796, C > T*	67.8 (22.9–234.9)	1.1 × 10^− 23^	287.6 (37.6–120 34.6)	1.5 × 10^− 18^	47.6 (12.8–219.8)	1.5 × 10^− 12^	20.9 ( 3.3–164.6)	3.2 × 10^− 4^
*PSORS1C1* *rs2233945, C > A*	60.0 (20.6–203.6)	9.7 × 10^− 23^	287.6 (37.4–12034.6)	1.5 × 10^− 18^	37.9 (10.6–160.2)	1.8 × 10^− 11^	20.9 ( 3.3–164.6)	3.2 × 10^− 4^
*PSORS1C1* *rs9263726, G > A*	67.8 (22.9–234.9)	1.1 × 10^− 23^	287.6 (37.6–12034.6)	1.5 × 10^− 18^	47.6 (12.8–219.8)	1.5 × 10^− 12^	20.9 ( 3.3–164.6)	3.2 × 10^− 4^
*TCF19* *rs1044870, C > T*	Inf (7.3 - Inf)	2.0 × 10^− 7^	Inf (0.8 - Inf)	3.8 × 10^− 2^	Inf (9.3 - Inf)	6.2 × 10^− 7^	Inf (11.4 - Inf2)	1.3 × 10^− 5^
*TCF19* *rs9263794, A > G*	43.53 (15.9–134.2)	5.3 × 10^− 21^	165.81 (23.3–7019.3)	6.9 × 10^− 16^	35.2 (9.7–166.5)	2.1 × 10^− 11^	11.87 (2.0–86.3)	2.2 × 10^− 3^

*Abbreviations* cutaneous adverse drug reactions, cADRs; Stevens - Johnson syndrome, SJS; toxic epidermal necrolysis, TEN; drug reaction with eosinophilia and systemic symptoms, DRESS; maculopapular exanthema, MPE; 95% confidence interval, 95% CI; and single nucleotide polymorphisms, SNPs

### Association of the combined SNPs with allopurinol-induced cADRs

To predict the risks for allopurinol-induced cADRs, combinations of *rs3099844* (*HCP5*), *rs9263726* (*PSORS1C1*), *rs9263733* (*POLR2LP*) and *rs9263745* (*CCHCR1*) were analyzed as shown in Table 4. Out of the 57 case patients, 51 (89.5%) carried variant alleles of these SNPs. It was found that allopurinol-induced cADRs were significantly higher in the case patients that carried these combined SNPs compared to the tolerant controls (OR 73.2; 95% CI 24.2-266.8; *P* = 1.9 × 10^− 24^). The percentage for sensitivity, specificity, PPV, and NPV were 84%, 94%, 9%, and 100%, respectively as shown in Supplement 3. It was further found that the risks for cADRs were driven from the SJS/TEN cases since the risks for allopurinol-induced SJS/TEN were significantly higher in 24/25 (96.0%) of the patients that carried these combined SNPs (*rs3099844, rs9263726, rs9263733*, and *rs9263745*) compared to the tolerant controls (OR 200.9; 95% CI 27.6-8484.4; *P* = 7.5 × 10^− 17^), as shown in Table 4. The percentages for sensitivity, specificity, PPV, and NPV were 71%, 99%, 31%, and 100%, respectively (Supplement 3). Allopurinol-induced DRESS was also significantly higher in 20 out of 24 (83.3%) of the patients that carried these combined SNPs (*rs3099844*, *rs9263726*, *rs9263733*, and *rs9263745*) compared to the tolerant controls (OR 42.9; 95%CI 11.5–208.9; *P* = 3.0 × 10^− 12^). The percentage for sensitivity, specificity, PPV, and NPV were 67%, 96%, 10%, and 100%, respectively (Supplement 3). The SNP combinations (*rs3099844*, *rs9263726*, *rs9263733*, and *rs9263745*) were detected in six out of eight (75.0%) of the allopurinol-induced MPE cases. The association between allopurinol-induced MPE and the combined SNPs was significant, with an OR of 25.7 (95% CI 3.9–291.3, *P* = 9.3 × 10^− 5^). The percentages for sensitivity, specificity, PPV, and NPV were 38%, 98%, 11%, and 100%, respectively, as shown in Supplement 3. The 4 SNPs haplotype showed significant association when compared between allopurinol-induced cADRs and tolerant controls. However, use of these four SNPs to predict susceptibility to cADRs did not seem to be as sensitive as HLA-B*58:01 typing since the OR was lower and the *p* value was higher.

**Table 4:** The association between the combined SNPs and allopurinol-induced cADRs vs. the tolerant controls

HLA/SNPs allele	cADRs vs. tolerant control		SJS-TEN vs. tolerant control		DRESS vs. tolerant control		MPE vs. tolerant control	
	Odds ratio (95%CI)	P-value	Odds ratio (95%CI)	P-value	Odds ratio (95%CI)	P-value	Odds ratio (95%CI)	P-value
HLA-B*58:01	137.2 (38.3–670.5)	1.7 × 10^− 27^	582.0 (62.2–5447.3)	3.7 × 10^− 23^	92.2 (22.6–375.1)	2.3 × 10^− 14^	63.7 (8.4–829.2)	2.7 × 10^− 6^
rs3099844, C > A rs9263726,G > A	53.1 (18.9–170.3)	2.2 × 10^− 22^	200.9 (27.6–8484.4)	7.5 × 10^− 17^	42.9 (11.5–208.9)	3.0 × 10^− 12^	3.4 (0.5–17.3)	1.1 × 10^− 1^
rs3099844, C > A rs9263726,G > A rs9263733, C > T	73.2 (24.2–266.8)	1.9 × 10^− 24^	200.9 (27.6–8484.4)	7.5 × 10^− 17^	42.9 (11.5–208.9)	3.0 × 10^− 12^	14.5 (2.4 -107.6)	1.1 × 10^− 3^
rs3099844, C > A rs9263726,G > A rs9263733, C > T rs9263745, G > A	73.2 (24.2–266.8)	1.9 × 10^− 24^	200.9 (27.6–8484.4)	7.5 × 10^− 17^	42.9 (11.5–208.9)	3.0 × 10^− 12^	25.7 (3.9–291.3)	9.3 × 10^− 5^
rs3099844, C > A rs9263726,G > A rs9263733, C > T rs9263745, G > A rs4084090, A > G	56.9 (18.9–214.6)	3.4 × 10^− 22^	129.9 (18.6–5505.9)	1.4 × 10^− 14^	38.3 (9.8–224.8)	2.0 × 10^− 11^	16.4 (2.6–181.7)	5.7 × 10^− 4^

*Abbreviations* cutaneous adverse drug reactions, cADRs; Stevens-Johnson syndrome, SJS; toxic epidermal necrolysis, TEN; drug reaction with eosinophilia and systemic symptoms, DRESS; maculopapular exanthema, MPE; SNPs, Single nucleotide polymorphisms; and 95% CI, 95% Confidence Interval

## Discussion

According to data from the Clinical Pharmacogenetics Implementation Consortium (CPIC) guidelines, HLA-B*58:01 allele has been identified as the genetics marker of allopurinol-induced SCARs in many ethnicities [1, 2, 5, 6]. Our study, we found the specific association of HLA-B*58:01 allele and allopurinol-induced cADRs (OR = 137.2, *P*-value = 1.7 × 10^− 27^), SJS-TEN (OR = 582.0, *P*-value = 3.7 × 10^− 23^), DRESS (OR = 92.2, *P*-value = 2.3 × 10^− 14^) and MPE (OR = 63.7, *P*-value = 2.7 × 10^− 6^) in Thai population. After analyzing the 15 SNPs in the 9 potential genes of interest, the current study established statistically significant strong associations between the combined 4 SNPs (*rs3099844*, *rs9263726*, *rs9263733*, and *rs9263745*) in 4 genes of interest (*HCP5*, *PSORS1C1*, *POLR2LP*, and *CCHCR1*) and allopurinol-induced cADRs in Thai patients. The findings of the current study suggested that these SNPs could be used as alternative novel biomarkers for predicting cADRs in patients taking allopurinol.

These findings are the first demonstration, to our knowledge, of an association between the SNPs located on the 6p21 region and allopurinol-induced cADRs in Thai patients. Our findings supported a prior GWAS in Japan that reported a significant association between *PSORS1C1* (*rs9263726*) located in 6p21 and allopurinol-induced SJS/TEN [13]. However, in the current study, we established a very strong association between allopurinol-induced cADRs and the combined four SNPs (*rs3099844*, *rs9263726*, *rs9263733*, and *rs9263745*). Moreover, the frequencies of *rs9263733* (*CT*, heterozygous), *rs3099844* (*CA*, heterozygous), *rs9263726* (*GA*, heterozygous) and *rs9263745* (*AA*, homozygous variants) were found in two of eight (25.0%), seven of eight (87.5%), one of eight (12.5%) and one of eight (12.5%), respectively, of the allopurinol-induced cADR patients who lacked the HLA-B*58:01 gene. This was the great superiority of the current investigation. These SNPs may be considered as potential novel biomarkers for predicting risks for allopurinol-induced cADRs. It is worth mentioning here that HLA-B*58:01 is a well-established biomarker used to predict SCARs. Since our study also included the MPE rashes, this may have caused a lower sensitivity of the test compared to previous studies conducted in Thailand. Although the association of *rs3099844* with SJS/TEN was previously noted in a Japanese population [14] and a Mozambique population [19], we proposed that all of the SNPs identified in our study would be related to skin diseases, e.g., SJS/TEN and psoriasis [20–22]. Furthermore, a functional analysis of these SNPs might be useful to determine the pathogenesis of allopurinol-induced cADRs, which would warrant further studies to establish the mechanistic pathways of these associations.

Previous study, that may provide insightful information about potentially recessive genetic variants present in the same or opposite alleles, and therefore, they might be helpful for diagnosings disease pathogenicity. Besides genetic factors, some non-genetic factors e.g., age and comorbidities such as chronic kidney disease (CKD), as well as the dose of allopurinol, should be taken into consideration to reduce the incidence of allopurinol-induced SCARs [23]. Non-genetic predispositions for allopurinol-induced SCARs include allopurinol dose and gender. Daily doses equal to or greater than 200 mg have been associated with higher risk (adjusted OR 36; 95% CI 17–76) compared to lower doses (adjusted OR 3.0; 95% CI 1.1–8.4) [24]. The American College of Rheumatology has conditionally recommended HLA–B*58:01 allele testing prior to starting allopurinol for patients of Southeast Asian descent (e.g., Han Chinese, Korean, or Thai) and for African American patients, over not testing for the HLA–B*58:01 allele. Universal testing for the HLA–B*58:01 allele prior to starting allopurinol is conditionally recommended against in patients of other ethnic or racial background over testing for the HLA–B*58:01 allele. As noted above, starting allopurinol at daily doses of ≤ 100 mg (and lower doses in patients with CKD) is strongly recommended over starting at higher doses [23]. Females have higher risks than males for developing allopurinol-induced SCARs. Moreover, advanced-age patients have high mortality rates when taking allopurinol. For the univariate analysis consisting of sex and age, we found that age had no effect with the SNPs, but females had higher risks than males, which aligned with previous study [25]. For the multivariate analysis, we found that both sex and age had no effects with the SNPs (Supplement 4).

Precision medicine treatment through pharmacogenomics testing could improve their prevention of drug-induced SCARs and increase treatment efficiency. Moreover, the cost of genetics testing is a major factor for determining the decision making in patients. Accordance with data from Bank of Thailand in 2023, the cost was converted at the rate of 35 THB per 1 USD (https://www.bot.or.th/th/statistics/exchange-rate.html). Recently, the cost data reported the pharmacogenomics testing of HLA-B*58:01 allele with PCR-SSOP technique (approximately 2,000 Thai Baht, (THB) and nearing USD 56.57). Similarly, the cost of multiple *SNPs* genotyping has fallen by Real-time PCR technique and the cost of approximately USD 57 from Pharmacogenomics and Personalized Medicine (PPM), Ramathibodi Hospital, Mahidol University. Even though, cost of the pharmacogenomics testing is a one of the barriers to implement pharmacogenomics in clinical practice. It is generally assumed that multi-gene testing might have better clinical utility compared to single-gene testing if such testing is cost-effective. Interestingly, the cost of these combined *rs3099844, rs9263726, rs9263733*, and *rs9263745* SNPs are almost identical to HLA-B*58:01. There are several molecular methods for HLA-B*58:01 genotyping such as sequence specific oligonucleotide probe hybridization (SSO), sequence-specific primers polymerase chain reaction (SSP). In this study, we proposed the rapid and reliable assay for HLA-B*58:01 identification prior to allopurinol administration by detection of haplotype of 4 SNPs (*rs3099844*, *rs9263726*, *rs9263733*, and *rs9263745*) to identify the risk patient of allopurinol-induced SCARs in clinical settings. With the presence of these SNPs, further studies should analyse the cost-effectiveness and availability of multi-gene testing for better comprehension.

There were some strengths and limitations in our analyses. HLA-B*58:01 is a well-established biomarker for allopurinol-induced SCARs in Thai populations. We observed similar prevalence rates for the variant alleles of the SNP combinations. Therefore, we suggest that any patient carrying the high-risk alleles of these combined SNPs will be at risk for developing allopurinol-induced cADRs. The molecular mechanisms leading to allopurinol-related cADRs associated with these SNPs should be investigated in future studies to support our findings. It is also important to confirm the above findings with other independent data for relatively larger sample sizes of Thai populations, as well as for other ethnic groups.

In conclusion, this our research confirms the specific association between HLA-B*58:01 and allopurinol-induced CADRs including SJS-TEN, DRESS and MPE in Thais. Furthermore, there was a strong statistically significant association between the combined four SNPs (*rs3099844*, *rs9263726*, *rs9263733*, and *rs9263745*) and allopurinol-induced cADRs. The findings of the current study suggest that these SNPs could be used as alternative novel biomarkers for predicting cADRs in patients taking allopurinol, especially in settings where normal *HLA* typing is unavailable. Further investigations into the economics and functions of these SNPs could be elucidated in future studies for broader clinical applications.

### Electronic supplementary material

Below is the link to the electronic supplementary material.

Supplementary Material 1

Supplementary Material 2

Supplementary Material 3

Supplementary Material 4

## Data Availability

The datasets and materials presented in this study can be found in the main manuscript and the supplementary material, and they may be obtained from the corresponding author on reasonable request.
