# Supplementary material for: Influence of pharmacogenomic polymorphisms on allopurinol-induced cutaneous adverse drug reactions in Thai patients
Source: BMC Med Genomics. 2024 Apr 23;17:101. doi: 10.1186/s12920-024-01874-y (PMC11040848; doi:10.1186/s12920-024-01874-y)
Supplement: Supplementary file 1 — Supplementary Material 1 [file 12920_2024_1874_MOESM1_ESM.docx]

**Supplement 1.** The percentage of sensitivity, specificity, PPV, and NPV of SNPs

| **Gene,**  **SNPs, allele** | **Sensitivity (%)** | | | | **Specificity (%)** | | | | **PPV (%)** | | | | **NPV (%)** | | | |
| --- | --- | --- | --- | --- | --- | --- | --- | --- | --- | --- | --- | --- | --- | --- | --- | --- |
|  | **cADRs** | **SJS-TEN** | **DRESS** | **MPE** | **cADRs** | **SJS-TEN** | **DRESS** | **MPE** | **cADRs** | **SJS-TEN** | **DRESS** | **MPE** | **cADRs** | **SJS-TEN** | **DRESS** | **MPE** |
| *HLA-B*5801* | 86 | 96 | 83 | 75 | 96 | 96 | 96 | 96 | 92 | 86 | 83 | 60 | 92 | 99 | 95 | 93 |
| *93BAT1* rs2734583, A>G | 77 | 88 | 79 | 38 | 93 | 93 | 93 | 93 | 86 | 76 | 73 | 30 | 88 | 97 | 95 | 95 |
| *BAT3* rs3117583, A>G | 67 | 80 | 58 | 50 | 84 | 84 | 84 | 84 | 70 | 56 | 47 | 20 | 82 | 94 | 89 | 96 |
| *CCHCR1 rs130077, G>A* | 84 | 96 | 79 | 63 | 93 | 93 | 93 | 93 | 87 | 77 | 73 | 42 | 91 | 99 | 95 | 97 |
| *CCHCR1 rs9263745, G>A* | 86 | 96 | 79 | 75 | 93 | 93 | 93 | 93 | 88 | 77 | 73 | 46 | 92 | 99 | 95 | 98 |
| *CCHCR1 rs9263785, T>G* | 84 | 96 | 79 | 63 | 93 | 93 | 93 | 93 | 87 | 77 | 73 | 42 | 91 | 99 | 95 | 97 |
| *HCP5 rs3099844, C>A* | 79 | 88 | 83 | 50 | 92 | 92 | 85 | 85 | 85 | 73 | 57 | 21 | 89 | 97 | 96 | 96 |
| *HCP5 rs3131643, G>A* | 81 | 88 | 83 | 38 | 85 | 85 | 92 | 92 | 75 | 59 | 71 | 27 | 89 | 97 | 96 | 95 |
| *HLAC rs4084090, A>G* | 86 | 96 | 83 | 63 | 89 | 89 | 89 | 89 | 82 | 69 | 65 | 31 | 92 | 99 | 96 | 97 |
| MSH5 rs1150793, A>G | 67 | 80 | 58 | 50 | 82 | 81 | 81 | 81 | 68 | 51 | 42 | 17 | 81 | 94 | 89 | 95 |
| *POLR2LP* rs9263733, C>T | 84 | 96 | 79 | 63 | 95 | 95 | 95 | 95 | 91 | 83 | 79 | 50 | 91 | 99 | 95 | 97 |
| *POU5F1* rs9263796, C>T | 84 | 96 | 79 | 63 | 93 | 93 | 93 | 93 | 87 | 77 | 73 | 42 | 91 | 99 | 95 | 97 |
| *PSORS1C1 rs2233945, C>A* | 82 | 96 | 75 | 63 | 93 | 93 | 93 | 93 | 87 | 77 | 72 | 42 | 90 | 99 | 94 | 97 |
| *PSORS1C1 rs9263726, G>A* | 84 | 96 | 79 | 63 | 93 | 93 | 93 | 93 | 87 | 77 | 73 | 42 | 91 | 99 | 95 | 97 |
| *TCF19 rs1044870, C>T* | 25 | 8 | 33 | 50 | 100 | 100 | 100 | 100 | 100 | 100 | 100 | 100 | 70 | 81 | 86 | 96 |
| *TCF19 rs9263794, A>G* | 86 | 96 | 83 | 63 | 88 | 88 | 88 | 88 | 80 | 67 | 63 | 29 | 92 | 99 | 96 | 97 |

^cutaneous adverse drug reactions, cADRs; Stevens-Johnson syndrome, SJS; toxic epidermal necrolysis, TEN; drug reaction with eosinophilia and systemic symptoms, DRESS; maculopapular exanthema, MPE;^ ^Negative predictive value, NPV; Positive predictive value, PPV and SNPs, Single nucleotide polymorphisms.^
