# Supplementary material for: Influence of pharmacogenomic polymorphisms on allopurinol-induced cutaneous adverse drug reactions in Thai patients
Source: BMC Med Genomics. 2024 Apr 23;17:101. doi: 10.1186/s12920-024-01874-y (PMC11040848; doi:10.1186/s12920-024-01874-y)
Supplement: Supplementary file 2 — Supplementary Material 2 [file 12920_2024_1874_MOESM2_ESM.docx]

**Supplement 2.** Allopurinol-induced cADRs patients without *HLA-B*58:01* allele

| **No** | **cADRs** | ***HLA-B***  **genotype** | ***rs9263726*** | ***rs2233945*** | ***rs9263733*** | ***rs9263745*** | ***rs130077*** | ***rs9263785*** | ***rs9263794*** | ***rs9263796*** | ***rs4084090*** | ***rs3099844*** | ***rs2734583*** |
| --- | --- | --- | --- | --- | --- | --- | --- | --- | --- | --- | --- | --- | --- |
| 1 | DRESS | *40:01/46:01* | *GG* | *CC* | *CC* | *GG* | *GG* | *TT* | *AA* | *CC* | *AA* | *CC* | *AA* |
| 2 | MPE | *13:01/54:01* | *GG* | *CC* | *CC* | *GG* | *GG* | *TT* | *AA* | *CC* | *AA* | *CC* | *AA* |
| 3 | DRESS | *44:03/51:02* | *GG* | *CC* | *CC* | *GG* | *GG* | *TT* | *AG* | *CC* | *AG* | *CC* | *AA* |
| 4 | DRESS | *13:01/46:01* | *GG* | *CC* | *CC* | *GG* | *GG* | *TT* | *AA* | *CC* | *AA* | *CC* | *AA* |
| 5 | MPE | *15:02/40:01* | *GG* | *CC* | *CC* | *GG* | *GG* | *TT* | *AA* | *CC* | *AA* | *CC* | *AA* |
| 6 | SJS/TEN | *40:01/54:01* | *GG* | *CC* | *CC* | *GG* | *GG* | *TT* | *AA* | *CC* | *AA* | *CC* | *AA* |
| 7 | DRESS | *18:01:51:01* | *GG* | *CC* | *CC* | *GG* | *GG* | *TT* | *AA* | *CC* | *AA* | *CC* | *AA* |
| 8 | DRESS | *13:02/38:02* | *AG* | *AC* | *CT* | *AA* | *AG* | *GT* | *AG* | *CT* | *AG* | *AC* | *AG* |

cADRs; Stevens-Johnson syndrome, SJS; toxic epidermal necrolysis, TEN; drug reaction with eosinophilia and systemic symptoms, DRESS; maculopapular exanthema, MPE; *PSORS1C1.rs9263726* (G>A), *PSORS1C1_rs2233945* (C>A), *POLR2LP_rs9263733* (C>T), *CCHCR1_rs9263745* (G>A), *CCHCR1_rs130077* (G>A), *CCHCR1_rs9263785* (T>G), *TCF19_rs9263794* (A>G), *POU5F1_rs9263796* (C>T), *HLA-C*_rs4084090 (A>G), *HCP5_rs3099844* (C>A) and *BAT1-rs2734583* (A>G)
