# Supplementary material for: Influence of pharmacogenomic polymorphisms on allopurinol-induced cutaneous adverse drug reactions in Thai patients
Source: BMC Med Genomics. 2024 Apr 23;17:101. doi: 10.1186/s12920-024-01874-y (PMC11040848; doi:10.1186/s12920-024-01874-y)
Supplement: Supplementary file 3 — Supplementary Material 3 [file 12920_2024_1874_MOESM3_ESM.docx]

**Supplement 4.** Univariate and multivariate analysis

| **Gene, SNP** | **Sex** | | | **Age** | | | **Multivariate (Age & Sex)** | | |
| --- | --- | --- | --- | --- | --- | --- | --- | --- | --- |
|  | **Odds Ratio** | **P-value** | **95%CI** | **Odds Ratio** | **P-value** | **95%CI** | **Odds Ratio** | **P-value** | **95%CI** |
| *BAT1*, rs2734583, A>G | 1.86 | 0.09 | 0.91-3.78 | 1.00 | 0.98 | 0.97-1.02 | 0.48 | 0.33 | 0.11-2.09 |
| *HCP5*,rs3099844, C>A | 2.24 | 0.02 | 1.12-4.47 | 1.01 | 0.65 | 0.98-1.03 | 0.44 | 0.27 | 0.10-1.90 |
| PSORS1C1_rs9263726, G>A | 2.67 | 0.01 | 1.33-5.39 | 1.01 | 0.31 | 0.99-1.03 | 0.27 | 0.09 | 0.06-1.21 |
| PSORS1C1_rs2233945, C>A | 2.27 | 0.02 | 1.13-4.58 | 1.01 | 0.49 | 0.98-1.03 | 0.32 | 0.14 | 0.07-1.44 |
| POLR2LP, rs9263733, C>T | 2.27 | 0.02 | 1.13-4.58 | 1.01 | 0.48 | 0.98-1.03 | 0.32 | 0.14 | 0.07-1.44 |
| CCHCR1, rs9263745, G>A | 2.46 | 0.01 | 1.22-4.97 | 1.01 | 0.28 | 0.99-1.04 | 0.30 | 0.12 | 0.07-1.36 |
| CCHCR1, rs130077, G>A | 2.46 | 0.01 | 1.22-4.97 | 1.01 | 0.42 | 0.99-1.04 | 0.30 | 0.12 | 0.07-1.36 |
| CCHCR1, rs9263785, T>G | 2.46 | 0.01 | 1.22-4.97 | 1.01 | 0.42 | 0.99-1.04 | 0.30 | 0.12 | 0.07-1.36 |
| TCF19, rs9263794, A>G | 2.43 | 0.01 | 1.21-4.85 | 1.02 | 0.19 | 0.99-1.04 | 0.24 | 0.06 | 0.05-1.08 |
| TCF1, rs1044870, C>T | 0.63 | 0.50 | 0.17-2.4 | 1.02 | 0.32 | 0.98-1.06 | 0.03 | 0.01 | 0.001-0.46 |
| POU5F1, rs9263796, C>T | 1.16 | 0.67 | 0.59-2.29 | 1.00 | 0.65 | 0.98-1.03 | 0.79 | 0.74 | 0.19-3.15 |
| HLAC, rs4084090, A>G | 2.53 | 0.01 | 1.26-5.08 | 1.01 | 0.20 | 0.99-1.04 | 0.24 | 0.07 | 0.05-1.09 |
| HCP5_rs3131643, G>A | 1.55 | 0.21 | 0.78-3.06 | 1.01 | 0.29 | 0.99-1.03 | 0.34 | 0.14 | 0.087-1.41 |
| BAT3, _rs3117583, A>G | 1.68 | 0.15 | 0.84-3.38 | 1.02 | 0.11 | 0.99-1.04 | 0.19 | 0.04 | 0.04-0.90 |
| MSH5, _rs1150793, A>G | 1.91 | 0.07 | 0.95-3.84 | 1.03 | 0.02 | 1.00-1.05 | 0.11 | 0.01 | 0.02-0.55 |

95% CI, 95% Confidence Interval; SNPs, Single nucleotide polymorphisms.
