# Supplementary material for: Influence of pharmacogenomic polymorphisms on allopurinol-induced cutaneous adverse drug reactions in Thai patients
Source: BMC Med Genomics. 2024 Apr 23;17:101. doi: 10.1186/s12920-024-01874-y (PMC11040848; doi:10.1186/s12920-024-01874-y)
Supplement: Supplementary file 4 — Supplementary Material 4 [file 12920_2024_1874_MOESM4_ESM.docx]

Supplement 3. The percentage of sensitivity, specificity, PPV, and NPV of Combine SNPs*

| SNP, allele | Sensitivity (%) | | | | Specificity (%) | | | | PPV (%) | | | | NPV (%) | | |  |
| --- | --- | --- | --- | --- | --- | --- | --- | --- | --- | --- | --- | --- | --- | --- | --- | --- |
|  | cADRs | SJS-TEN | DRESS | MPE | cADRs | SJS-TEN | DRESS | MPE | cADRs | SJS-TEN | DRESS | MPE | cADRs | SJS-TEN | DRESS | MPE |
| *HLA-B*58:01* | 86 | 96 | 83 | 75 | 96 | 96 | 96 | 96 | 92 | 86 | 83 | 60 | 92 | 99 | 95 | 93 |
| rs3099844, C>A rs9263726,G>A | 83 | 71 | 67 | 33 | 92 | 99 | 96 | 97 | 7 | 31 | 10 | 7 | 100 | 100 | 100 | 100 |
| rs3099844, C>A rs9263726,G>A rs9263733, C>T | 84 | 71 | 67 | 33 | 94 | 99 | 96 | 97 | 9 | 31 | 10 | 7 | 100 | 100 | 100 | 100 |
| rs3099844, C>A rs9263726,G>A rs9263733, C>T rs9263745, G>A | 84 | 71 | 67 | 38 | 94 | 99 | 96 | 98 | 9 | 31 | 10 | 11 | 100 | 100 | 100 | 100 |
| rs3099844, C>A rs9263726,G>A rs9263733, C>T rs9263745, G>A rs4084090, A>G | 78 | 62 | 58 | 29 | 95 | 99 | 97 | 98 | 9 | 27 | 11 | 8 | 100 | 100 | 100 | 99 |

cutaneous adverse drug reactions, cADRs; Stevens-Johnson syndrome, SJS; toxic epidermal necrolysis, TEN; drug reaction with eosinophilia and systemic symptoms, DRESS; maculopapular exanthema, MPE; Negative predictive value, NPV; Positive predictive value, PPV and SNPs, Single nucleotide polymorphisms.
